# Digital PCR: What Relevance to Plant Studies?

**DOI:** 10.3390/biology9120433

**Published:** 2020-11-30

**Authors:** Caterina Morcia, Roberta Ghizzoni, Chiara Delogu, Lorella Andreani, Paola Carnevali, Valeria Terzi

**Affiliations:** 1 Consiglio per la Ricerca in Agricoltura e L’analisi Dell’economia Agraria-Centro di Ricerca Genomica e Bioinformatica (CREA-GB), via San Protaso 302, 29017 Fiorenzuola d’Arda, Italy; caterina.morcia@crea.gov.it (C.M.); roberta.ghizzoni@crea.gov.it (R.G.); 2 Consiglio per la Ricerca in Agricoltura e L’analisi Dell’economia Agraria-Centro di Ricerca Difesa e Certificazione (CREA-DC), via Emilia km 307, 26838 Tavazzano, Italy; chiara.delogu@crea.gov.it (C.D.); lorella.andreani@crea.gov.it (L.A.); 3 Barilla S.p.A., via Mantova 166, I-43122 Parma, Italy; paola.carnevali@barilla.com

**Keywords:** digital PCR, genetic traceability, diagnostics, genetically modified organisms, species, copy number variation, gene expression

## Abstract

**Simple Summary:**

Digital PCR is a third-generation technology based on the subdivision of the analytical sample into numerous partitions that are amplified individually. This review presents the major applications of digital PCR (dPCR) technology developed so far in the field of plant science. In greater detail, dPCR assays have been developed to trace genetically modified plant components, pathogenic and non-pathogenic microorganisms, and plant species. Other applications have concerned the study of the aspects of structural and functional genetics.

**Abstract:**

Digital PCR (dPCR) is a breakthrough technology that able to provide sensitive and absolute nucleic acid quantification. It is a third-generation technology in the field of nucleic acid amplification. A unique feature of the technique is that of dividing the sample into numerous separate compartments, in each of which an independent amplification reaction takes place. Several instrumental platforms have been developed for this purpose, and different statistical approaches are available for reading the digital output data. The dPCR assays developed so far in the plant science sector were identified in the literature, and the major applications, advantages, disadvantages, and applicative perspectives of the technique are presented and discussed in this review.

## 1. Introduction

**Figure 1:**
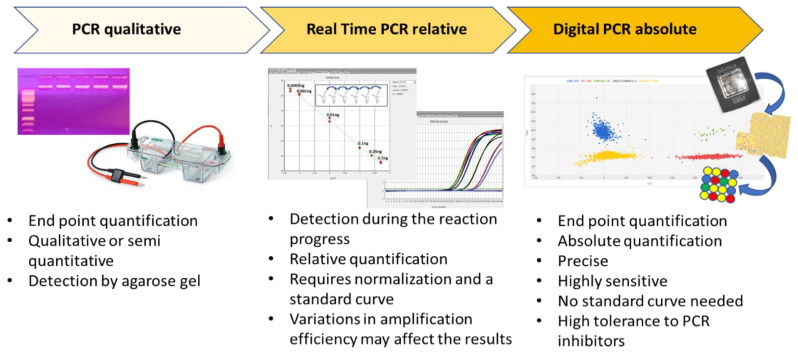
The three main PCR technologies are reported, and their main features are schematically presented.

Digital PCR (dPCR) is a breakthrough technology that is able to provide sensitive and absolute nucleic acid quantification [1]. It is a third-generation technology in the field of nucleic acid amplification. Starting from end-point PCR, which is able to provide a qualitative or semi-quantitative result, the second-generation of the technique—real time PCR or qPCR—gives a quantification of the target sequence (Figure 1). Digital PCR, the third-generation PCR technology, works by partitioning a sample of DNA or cDNA into a high number of single, parallel PCR reactions. The reactions are carried out in separated and numerous small volume compartments in which DNA or cDNA molecules of the sample are randomly distributed. Each compartment can host none, one, or many molecules. In ideal conditions (i.e., a low density of target DNA and a high number of compartments), each compartment holds one or none target molecule. In such conditions, after amplification and absorbance measurements, a compartment containing no target molecule is counted as 0, whereas a compartment with one target is counted as 1. From such data, considering the dilution factor, it is possible to calculate the target copy number in an analytical sample. However, because a compartment can contain more than one target molecule in practice, correction factors based on Poisson statistical distribution—used to account for the probability of a partition initially containing more than one target—is used to achieve the final outcomes.

The first pioneering achievement of this technique was realized in 1992 by Sykes et al. [2], who used limiting dilutions of their samples and end-point signal quantifications for gene mutation detection. A milestone for dPCR technology development was the study of Vogelstein and Kinzler [3], dated to 1999. These authors amplified individual molecules of a sample in parallel PCRs with fluorescent probes, therefore transforming the exponential, analog nature of the PCR into a linear, digital signal. With the introduction of microfluidics, proposed by Liu et al. [4] in 2003, a major improvement was completed, and the hitherto dormant technique became of considerable interest [5]. The partition of DNA samples was done with the aid of micropumps and microvalves, thus increasing the accuracy of the dilution step. In 2011, droplet digital PCR (ddPCR) was proposed by Quantalife Co, LT, as a cheaper approach in comparison to the microfluidic one.

Currently, several different platforms are used for PCR reaction compartmentalization, such as active partitioning platforms (based on mechanical aid for the compartment formation), passive partitioning platforms (based on fluidic effects to create sub-volumes), self-digitization platforms (which combine both passive filling and partitioning), and droplet-based platforms (in which aqueous droplets act as microreactors). The main differences among platforms are in the number of partitions and in the number of samples that can be processed in a run.

In dPCR, the detection of the target is achieved similarly to in qPCR, with two main families of chemistries: DNA intercalating dyes and hydrolysis-based probes. As in qPCR, there is the possibility to organize multiplex reactions to simultaneously follow more than one target.

**Figure 2:**
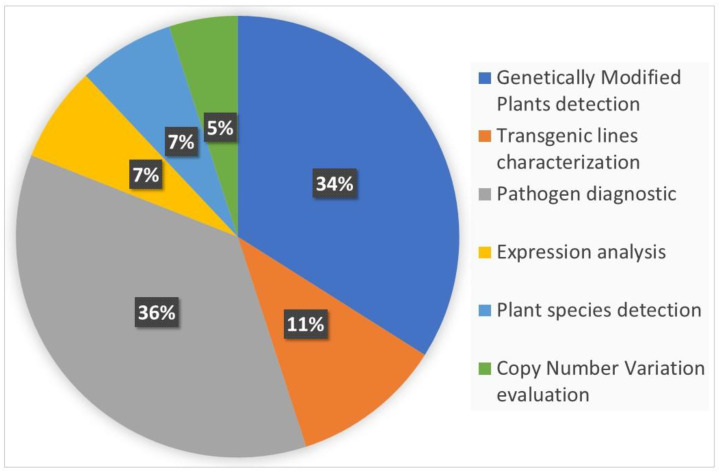
The main applications of digital PCR (dPCR) developed so far in the plant science sector are reported and classified into six main categories. The percentage of each category was calculated on the basis of number of recently published peer-reviewed studies. The literature screening was carried out using dPCR and its variants as main key words for an analysis of the recently published literature.

In 2004, Gachon et al. [6] published a review entitled “Real-time PCR: What Relevance to Plant Studies?” in which the “detection and quantification of foreign DNA” and the “quantification of specific transcripts” were reported as the major applications of the technology to the plant sector. Up to now, qPCR has had innumerable applications in the field of plant science, as expected. Now, dPCR has been proposed as a new technique applicable to the same targets of qPCR that is also able to provide answers to additional, biological questions. In the present review, we summarize the recent applications of dPCR in plant science. The literature of the last five years was screened, and 81 papers published in peer-reviewed international journals were found to present the development and application of new dPCR-based protocols. Figure 2 shows the main categories of dPCR applications and their percentages: more than 80% of the studies were focused on the detection and quantification of genetically modified plants (GMPs) and of plant pathogens, but other kinds of applications, such as plant species traceability, gene expression studies, and CNV (copy number variation) detection, have also been present.

## 2. Genetically Modified Plants Detection

More than twenty years after the start of their commercialization, biotech crops currently cover around 200 million hectares worldwide [7]. Different labeling laws and voluntary labelling systems have been put into effect by various countries and groups with the aim of informing the consumer about the GMP content in products intended for consumption. Consequently, numerous analytical assays have been developed and validated over time with the aim of identifying and quantifying transgenic components in the various agri-food chains. A range of DNA-based methodologies have been developed using, among others, PCR, arrays, sequencing, and biosensor technologies. Currently, most of the validated assays are based on the qPCR method based on TaqMan probes. However, this analytical sector has shown strong interest in the adoption of dPCR assays, as reviewed by Demeke and Dobnik [8]. From the analysis of the literature of the last years, it is evident that a major sector in which dPCR has found application is the identification of GMP components in raw materials, as well as in derived food and feed. Thirthy-four percent of the published studies considered in this review were in fact focused on this goal.

The starting point of dPCR assays for GMP detection is the availability of primers/probes targeting a specific transgenic sequence and of primers/probes targeting an endogenous gene of the plant. This latter is not the modified gene. It is a single-copy, native gene that is strongly conserved in the species object of the analysis. The right choice of such an endogenous sequence is of great significance because it provides vital information on the stability and reliability of the detection system and it permits the quantification of genetically modified ingredients in mixtures. The analytical pipeline for the quantification through dPCR of GM components is not substantially different from that for qPCR and can be summarized in the following modules:

- Sample preparation and DNA extraction.
- Digital PCR analysis for the amplification of the target transgenic sequence.
- Digital PCR analysis for amplification of a native, reference sequence.
- Data evaluation.

The ratio between transgene copy number and reference gene copy number provides the GM percentage present in a sample. However, EU legislation requires that the amount of GM content is estimated as a mass fraction, and a conversion factor was therefore established for each event to convert a copy number ratio into a mass fraction [9]

Table 1 summarizes some of the dPCR assays recently developed or evaluated for GMP traceability. In addition to the used instrumental platform and the plant species, the table reports the alfa-numeric identifier in the cases of authorized genetically modified lines (not available in case of not yet authorized experimental lines). Moreover, the plant endogenous gene(s) used as reference in the assay is reported.

Digital PCR approaches for transgene detection and quantification have been evaluated in cultivated GM crop and reference materials.

Various relevant points can be highlighted from the experiences made on dPCR-based GMP detection.

Iwobi et al. [12] compared qPCR and ddPCR performance on a panel of certified reference materials and GM samples arising from previous proficiency tests, and they demonstrated the applicability of ddPCR assays for the routine analysis of GM food and feed. Moreover, they demonstrated the better performance of ddPCR in comparison with qPCR in inhibitor-contaminated samples. The same results were obtained by Wang et al. [13] looking for transgenic rice in various processed samples. Other authors underlined the importance of selecting appropriate endogenous reference genes characterized by amplification efficiencies closely related to that of event-specific genes [11,16,20,34].

Several dPCR assays have been validated through proficiency studies in comparison with qPCR, and, in general, a similar performance has been found between the two techniques [12,18,19,21]. It has been found that sample pre-treatment prior to digital PCR can influence the results, and pretreatment-free detection is therefore preferential for achieving accurate results [26,31]. The convenience of digital PCR in real-life routine diagnostics has been verified on certified materials and analytical samples, and it has been found to useful for the routine quantification of GMP content in food and feed samples [12,23,27]. Multiplex assays for the quantification of several GM *Glycine max* lines were developed by Kosir et al. [30] and validated with an inter-laboratory trial. The assays performed well for key parameters such as the accuracy, robustness, and practicability and significant improvement in terms of cost efficiency has been demonstrated. The multiplexing of the assays therefore has the potentiality for further improvements of dPCR-based GMP traceability [32].

In conclusion, dPCR has several positive characteristics, such as its precision, accuracy even at very low target concentrations, suitability for routine analytics, and lower sensitivity to PCR inhibitors [8]. However, the main reason for the interest in this technique for GMP detection is its useful ability to perform an absolute quantification, independent of a reference standard/calibration curve. This means that differences in amplification efficiency due to matrix differences between a sample and its reference material do not affect the analytical results.

## 3. Transgenic Lines Characterization

A number of publications have reported the use of dPCR for the characterization of transgenic plants, here understood as experimentally transformed lines not proposed for cultivation and marketing. In this case, therefore, dPCR has a distinct use compared to what was reported in the previous paragraph. The purpose of dPCR analysis is, in fact, in this case not directed to the identification of transgenic components in food and feed but rather to the description and characterization of experimentally obtained transformed plants. In transformation projects, in fact, it is central to verify the success of transformation and to assess the inserted transgene copy number. Transformed lines carrying a single, full-length copy of the transgene are usually desirable to ensure a Mendelian inheritance of the transgene and to avoid silencing problems. Southern blot and qPCR analyses have been traditionally used to do such characterizations. However, the first technique is expensive and very laborious, and the second lacks the accuracy and precision needed to confidently characterize low copy number events. On the contrary, dPCR is emerging as a technology that is able to accurately identify transgene copy numbers, to discriminate between single and low transgene copies, and to do such characterization even in transgenic plants with large genomes. Moreover, the technique has been evaluated even for the determination of genome editing rates in CRISP/Cas9 (clustered regularly interspaced short palindromic repeats/associated protein 9) mutated plants [25]. Table 1 reports examples of dPCR applications to transformed plant characterization [19,20,23,28,33,37]. However, it is noteworthy that the published studies on this topic have apparently been much more numerous. The failure to enumerate other studies is probably due to the fact that in these works, the use of dPCR is not considered central and dPCR is therefore not included among the keywords, thus escaping the review. Beyond this limit on our part, it can be concluded, from the identified experimental works, that the shared opinion is that dPCR is a particularly useful for the fast and accurate characterization of transgenic lines, able to identify both homozygous and hemizygous individuals among large number of transformants.

CNV refers to genome structural variations in which a specific DNA segment is repeated, and the number of repeats varies among different genotypes. In polyploids, CNV can also be defined as variations in allele dosage at a locus. The different allele dosages can be correlated with different phenotypes, e.g., barley genotypes with an increased copy number of HvCBF4 and HvCBF2 transcription factors showed greater frost resistance [40]. Recently, CNV has been recognized as a key mechanism for plant evolution and crop domestication, as reviewed by Lye et al. [41]. Cytogenetic techniques, especially fluorescence in situ hybridization (FISH), have been traditionally used to study CNV. However, the FISH technique can be limited by the subjective evaluation of images and the fact that the procedure is technically demanding and expensive. Moreover, it can efficiently identify only copy number variants of thousands of base pairs. Other hybridization-based techniques, such as array comparative genome hybridization (aCGH) and SNP microarrays, have been developed to study CNV in plants. Next generation sequencing (NGS)-based methods have been proposed for CNV discovery, and computational approaches have been developed to optimize output data. Even qPCR has been applied to estimate differences in CNV, showing two main limitations, i.e., many replicates are required to achieve sensitive discrimination of differences in copy number and a relative measure rather than an absolute quantification is obtained.

The results obtained in the leading field of human genetics have indicated that dPCR could serve as a sensitive and less technically challenging method in respect to those above-reported to detect CNV. It is a particularly promising approach, both in diploid and polyploid plants. Zmienko et al. [42] used dPCR to study the CNV variation naturally present in three *Arabidopsis thaliana* loci and found strong variations in their copy numbers among natural ecotypes, demonstrating the high plasticity of the *Arabidopsis* genome. These authors compared two different analytical approaches for CNV determination, namely MLPA (multiplex ligation-dependent probe amplification) and ddPCR and concluded that ”dPCR should be the method of choice for discriminating loci with high copy numbers”. Two studies applied dPCR to the high-level polyploid sugarcane, a challenging crop species because of its complex genetics. McCord [43] positively evaluated the ability of dPCR to identify genotypes with high copy numbers of the *Bru1* gene, characterized by a higher level of resistance to sugarcane brown rust. The information gained with such assay is now used in USDA breeding programs. Sun and Joyce [20] developed three dPCR assays to be used for the determination of ploidy level and CNV in different *Saccharum* species. Jouanin et al. [44] tested the accuracy of dPCR for the CNV analysis of the alfa-gliadin gene family in *Triticum aestivum.* The authors concluded that dPCR is suitable for the high-throughput screening of gene-edited and mutated wheat plants.

## 4. Expression Analysis and Regulation

Several technologies, such as real-time PCR, sequencing, and hybridization-based assays, are currently used for gene expression studies. The suitability of digital PCR for gene expression analysis has been demonstrated in several plant species, such as *Amaranthus cruentus* [45], *Beta vulgaris* [46], *Castanea* [47], and *Hordeum vulgare* [48]. The expression of genes involved in resistance to pathogens, in response to bioactive compounds, in starch synthesis, and in senescence developmental process have been considered. An increasing application of dPCR can be hypothesized in the field of cis-regulatory elements and epigenetics. In the sector of regulatory molecules, the more relevant role of dPCR can be in the quantification of long non-coding RNA regions and microRNAs. In this case, in fact, there is no need for standard or endogenous controls, and no competition between different targets can arise. Consequently, dPCR can ensure precise microRNA quantification and even the possibility to identify rare sequences. Interestingly, dPCR has been even applied to track dietary microRNAs. The levels of a panel of *A. thaliana* microRNAs were evaluated in different *Plutella xylostella* tissues with the aim to study the cross-kingdom functions of microRNAs derived from host plants in insect herbivores [49].

## 5. Plant Species Traceability

Plant species traceability in food and feed chains can play a pivotal role in defending quality and safety. A dPCR assay is available to quantify *Triticum aestivum* and to differentiate this species from *Triticum durum*. This analytical tool has a great practical value in the Italian pasta production chain: the use of *Triticum durum* is, in fact, mandatory for pasta production, and *Triticum aestivum* is considered a contaminant whose percentage cannot exceed a maximum level of 3%. The assay developed by Morcia et al. [50] was demonstrated to suitable for *T. aestivum* traceability along the whole pasta production chain from grains to pasta. Digital PCR has been used as a tool to evaluate the quality and amplifiability of DNA extracted from a challenging matrix, such as olive oil [51]. Assays to quantify apricot kernels in marzipan and kidney beans in lotus seed paste were proposed, respectively, by Koppel et al. [52] and Dong et al. [53]. Starch used in the food industry can be obtained from different plant sources, such as potato, cassava, corn, and wheat. Additionally, depending on the raw materials used, starch can have different commercial values. Cassava starch is the main material in adulteration because of its lower price. To counteract potential fraud, Chen et al. [54] developed a dPCR assay for the specific detection of this plant species in starch products. Finally, to meet the need for quantification of allergens, a droplet digital PCR approach was shown to be reliable and sensitive enough to quantify the very common allergen soy in food [55].

## 6. Phytopathogens Diagnostics

dPCR is becoming an important new tool for use in the plant pathogen diagnostics and crop protection. Many examples of diagnostic assays ex novo developed or translated from similar qPCR assays have been recently published, as reported in Table 2. Several classes of pathogens have been targeted ranging from fungi and bacteria to viruses and phytoplasma. All the authors found several advantages over qPCR diagnostic assays, including absolute quantification without a standard curve, improved precision and accuracy, and more accurate quantitation. Moreover, the observed reduction of false negatives is critically important for the diagnosis of infections to be included in certification programs [56].

Interestingly, Dreo et al. [78] demonstrated how ddPCR assays can be successfully performed on pure bacterial suspensions without any previous DNA extraction. This means that it is possible to quickly establish a correlation between the target concentrations and the starting CFUs (colony forming units). Moreover, this means that dPCR is even suitable for the preparation of in-house reference materials, which is particularly important in the field of plant pathogen diagnostics, where no reference materials are commercially available. Reverse-transcription (RT) digital PCR has been applied for the absolute quantification of viruses and viroids [60,64], with the aim of identifying the main transmission routes, main transmission vectors, and the plants carrying latent infections—a step of great relevance in the control and certification of nurseries’ plants.

In addition to the identification of pathogens at the species level, dPCR can be applied to the identification of specific strains. An example is the study of Hua et al. [79], in which droplet digital PCR assays were developed to detect and characterize *Aspergillus* populations in soil with the aim to quantify the ratio between aflatoxigenic and atoxigenic strains. Zulak et al. [80] used dPCR to track two mutations in the *Blumeria graminis Cyp51* gene, which is able to confer high levels of resistance to triazoles in the field. The identification of such mutations in the pathogen field population has a key role in crop protection. In fact, the identification of resistant pathogenic strains carried out in an early stage of growth of the crop allows one to better modulate the consequent fungicidal treatments. Digital PCR has even been applied to the identification of non-pathogenic specific microorganism strains or populations present in the soil. A droplet digital PCR assay was developed by Xie et al. [81] for the quantitative detection of *Bacillus subtilis*, a typical plant growth-promoting bacterium, in rhizosphere samples. Its efficiency in the analysis of soil samples is one of the strengths of digital PCR that is considered less susceptible than qPCR to the PCR inhibitors present in DNA extracts. The success in DNA amplification is of paramount relevance for the soil samples [70] and dPCR can have a useful role in the study of environmental samples.

## 7. Other dPCR Applications

In addition to the macro categories discussed in the previous paragraphs, additional uses of the dPCR have been proposed both in applied and basic agricultural research. For example, dPCR has been applied to the single and/or multiple detection of SNPs linked to useful agronomic traits. An example in this sense is the study of Stevanato et al. [82], which aimed to quantitate an SNP able to distinguish between annual and biennial sugar beet flowering plants.

## 8. Minimum Information for Publication of Quantitative Digital PCR Experiments

In support of the scientific community that uses the dPCR, a dMIQE2020 (Minimum Information for Publication of Quantitative Digital PCR Experiments) was recently published by the dMIQE Group [83], and it is mainly intended to assist researchers in providing key experimental information and understanding of the associated experimental process. The first dMIQE guidelines were published in 2013 with the aim of improving the harmonization of dPCR results, data comparability, and reproducibility. The dMIQE guidelines provide a list of items to consider when publishing results from dPCR experiments. Such items can help in understanding, reproducing, and comparing dPCR results. The dMIQE guidelines therefore operate at the three levels: i) to ensure the replication of experiments, ii) to provide key information for researchers and reviewers to measure the technical quality of the analysis, and iii) to facilitate the harmonization of the reporting and comparison of the analytical data regardless of the dPCR instrumentation used. The adoption dMIQE2020, even by the scientific community involved in different plant science sectors, is a key point for the efficient use, exploitation, and further enhancement of dPCR technology.

## 9. Conclusions

In conclusion, digital PCR is, at least in the plant science sector, still a young but very promising technique.

The technology has many advantages and few disadvantages, which are summarized in Table 3. A disadvantage is that the assays can quantify within a narrower range of magnitude than qPCR. The major current limitation of the technique is its higher analytical costs and lower throughput compared to qPCR. Such disadvantages must be overcome with the refinement of technologies and the organization of multiplex reactions before dPCR competes with qPCR, which is now analytical labs’ workhorse. Pecoraro et al. [84] underlined that the advantages of duplex and multiplex dPCR assays are “cost efficiency, due to the fact that multiple standard curves are not needed. In addition, for assays where the GM target(s) and the reference gene are analysed in the same partition (droplet or chamber), possible pipetting errors are reduced when relative concentrations are calculated”.

Speaking of the advantages, some are unique to the dPCR and particularly strong. One is the greater resilience of the reaction to the presence of inhibitors, and this characteristic therefore makes the technique particularly valuable for the study of environmental and soil samples [85]. Moreover, in general, authors agree in affirming that dPCR exhibits greater precision than qPCR with an equivalent or higher sensitivity. This is a valuable feature in the support of other technologies, e.g., next generation sequencing for which dPCR quantification is sufficiently accurate in counting amplifiable library molecules to justify elimination of titration techniques [86].

The main advantage of dPCR, however, remains the fact that the analysis operates an absolute quantification that is free from the need for standard reference curves for the quantification of the target sequence. This characteristic is fundamental in various fields of plant science—in particular for GMP detection, for which the transition from qPCR assays to dPCR ones is often considered advantageous. The EURL GM FF (European Union Reference Laboratory for GM Food and Feed, hosted by Joint Research Center, Ispra, Italy) is maintaining a database of species-, element-, and event-specific qPCR assays (see GMOMETHODS: EU Database of Reference Methods for GMO (genetically modified organism) Analysis [87] for more information), many of which have been validated in collaborative trials and are in current use in GMO laboratories around the world. It has been demonstrated that such qPCR assays can be converted into dPCR ones with only minor modifications. Such a transition is positively considered because, in contrast to qPCR, no standard curve is needed for dPCR in GMO quantification. This peculiarity also makes the technique fundamental to support qPCR, the accuracy and commutability of which may be improved with the implementation of standards and calibrants quantified by dPCR. In agricultural and environmental application fields, in fact, it is not trivial to have reference materials to build standard reference curves.

In human genetics, the technique has experienced great development in the last ten years, progressing from an expensive and niche approach to a plethora of applications. In plant science, as previously reported, the main applications are currently observed in the two sectors of GMO traceability [80] and pathogen diagnostics. However, other applications, such as the analysis of gene expression and the determination of structural gene variants and mutations, are present. It should be noted that some assays have been developed for the determination of plant species but not of variety. Moreover, dPCR is a very promising technology for epigenetic studies, targeted to characterize long and short non-coding RNAs and chromatin regulators. In food and environmental science, dPCR will likely find more and more applications in ingredient traceability, microorganism detection, microbial population description, the calibration of standards, and the analysis of inhibitory samples.

## Figures and Tables

**Table 1:** The table shows recently developed dPCR assays aimed at identifying and quantifying genetically modified plants and at characterizing transgenic lines.

Instrumental Platform	Plant Species	Genetically Modified Line	Endogenous Reference Gene(s)	Bibliography
Chamber-based digital PCR	*Zea mays*	NK603, MON810, MON863, Bt176, 3272, MIR162, MIR604	*Adh* and *hmg*	[10]
Droplet digital PCR	*Oryza sativa*	Kefeng-6	*Sps2, RBE4,* and *ppi-PPF*	[11]
Droplet digital PCR	*Zea mays* *Glycine max*	MON88017, MON87460, MON89034, MIR162 CV127, MON87701, and MON87705	-	[12]
Droplet digital PCR	*Oryza sativa*	TT51-1	*PLD*	[13]
Droplet digital PCR	*Glycine max*	A2704-12, 356043, 305423, and 40-3-2	*Lec-1*	[14]
Droplet digital PCR	*Brassica napus* *Glycine max*	OXY235 DP305423	*hmg* *Lec-1*	[15]
Droplet digital PCR	*Brassica napus*	HCN92	*Cruciferin, CruA, FatA,* and *hmg-I/Y*	[16]
Droplet digital PCR	*Glycine max*	MON87769, MON87708, MON87705, FG72	*Lec-1*	[17]
Droplet digital PCR	*Glycine max, Zea mays*	RR, MON89788, 2704, Bt176, Bt11, MON810, GA21, NK603, MON863, 59122, MIR604, TC1507, and T25	*Lec-1* *hmg*	[18]
Droplet digital PCR	*Zea mays*	IE034	*Adh*	[19]
Droplet digital PCR	*Saccharum officinarum*	Q208 and Q240	*ACT*	[20]
Digital PCR	*Glycine max*	40-3-2, MON89788	*Lec*	[21]
Droplet digital PCR	*Zea mays*	DAS1507, DAS59122, GA21, MIR162, MIR604, MON810, MON863, MON89034, NK603, T25, Bt11, and MON88017	*hmgA*	[22]
Droplet digital PCR	*Oryza sativa, Citrus, Solanum tuberosum, Zea mays, Lycopersicon esculentum, Triticum*	Non-commercial plants	*Rice-OsUBC, citrus-CsDHN, potato-StAAP2, maize-ZmADH1, tomato-SISYS,* and *wheat-PINb-D1b*	[23]
Droplet digital PCR	*Lolium*	Non-commercial plant	*LpCul4*	[24]
Droplet digital PCR	*Medicago sativa*	Non-commercial plants	-	[25]
Chamber-based digital PCR Droplet digital PCR	*Zea mays*	GA21	*Adh1*	[26]
Droplet digital PCR	*Zea mays*	MON810	*hmg*	[27]
Droplet digital PCR	*Nicotiana tabacum*	Non-commercial plants	*Ntactin* and *NtTubulin_1*	[28]
Droplet digital PCR	*Solanum tuberosum*	AV43-6-G7	*fru*	[29]
Droplet digital PCR	*Glycine max*	15 lines (authorized or with valid EFSA application)	*Lec-1*	[30]
Chamber-based digital PCR Droplet digital PCR	*Zea mays*	MON810, MON863, TC1507, MIR604, MIR162, GA21, T25, NK603, and BT176	-	[31]
Droplet digital PCR	*Zea mays*	MON863, MON810, DP98140, MIR604, GA21, MON89034, and MIR162	*hmgA*	[32]
Droplet digital PCR	*Arabidopsis thaliana*	Non-commercial plant	*AAP1*	[33]
Droplet digital PCR	*Triticum*	Non-commercial plant	*ssII-D* and *waxy-D1*	[34]
Droplet digital PCR	*Zea mays*	Certified reference materials	*hmg*	[35]
Droplet digital PCR	*Glycine max*	multitarget DNA molecule encoding for eight transgene soy traits	*Lec-1*	[36]
Droplet digital PCR	*Brassica napus*	Non-commercial transgenic lines	*CruA*	[37]
Droplet digital PCR	*Glycine max*	DAS-68416-4	-	[38]
Droplet digital PCR	*Zea mays*	DAS1507 and NK603	*hmg* and *Adh1*	[39]

**Table 2:** The table shows recently developed dPCR assays aimed at identifying and quantifying plant pathogens.

Target Microorganism	Disease	Affected Crop	Reference
*Candidatus Liberibacter asiaticus*	Huanglongbing (HLB; yellow shoot disease)	*Citrus*	[56]
*Candidatus Liberibacter asiaticus*	Huanglongbing (HLB; yellow shoot disease)	*Citrus*	[57]
*Acidovorax citrulli*	Bacterial fruit blotch	*Cucurbitaceous*	[58]
Group 16SrIV phytoplasmas	Lethal yellowing (LY)	*Phoenix dactylifera*	[59]
*Apscaviroid* (apple chlorotic fruit spot viroid—ACFSVd)	Chlorotic fruit spots and bump-like symptoms on the skin of apples	*Malus*	[60]
*Xanthomonas citri* subsp. citri	Citrus bacterial canker	*Citrus*	[61]
Potato mop top virus	Potato mop top disease (tuber necrosis, internode reduction, foliar yellow spots, and plant chlorosis)	*Solanum tuberosum*	[62]
*Ilyonectria*	Black foot disease	*Vitis vinifera*	[63]
Citrus yellow vein clearing virus (CYVCV)	Yellow vein disease	*Citrus*	[64]
*Cadophora luteo-olivacea*	Petri disease and esca of grapevine	*Vitis vinifera*	[65]
*Fusarium graminearum, Fusarium culmorum, Fusarium sporotrichioides, Fusarium poae, Fusarium avenaceum*	Fusarium head blight	Small grain cereals	[66]
*Agrobacterium vitis*	Crown gall	*Vitis vinifera*	[67]
Pepino mosaic virus (PepMV)	Fruit marbling, leaf, and stem necrosis	*Lycopersicon esculentum*	[68]
*Aspergillus niger, Aspergillus welwitschiae, Aspergillus tubingensis, Aspergillus carbonarius*	Bunch rots and mycotoxin production	*Vitis vinifera*	[69]
*Phytophthora nicotianae*	Root rot, crown rot, fruit rot, leaf infection, and stem infection	*Nicotiana tabacum*	[70]
*Candidatus Phytoplasma asteris*	Aster yellows (AY)	*Brassica*	[71]
*Erwinia amylovora*	Fire blight	*Malus*	[72]
*Tilletia laevis*	Common bunt	*Triticum*	[73]
*Phytophtora infestans*	Late blight	*Solanum*	[74]
*Plasmodiophora brassicae*	Clubroot	*Brassica*	[75]
Potato virus Y strains	Mosaic symptoms	*Solanum*	[76]
*Spiroplasma citri*	Citrus stubborn disease	*Citrus*	[77]
*Erwinia amylovora* and *Ralstonia solanacearum*	Fire blight of rosaceous plants, potato brown rot	*Solanaceae, Rosaceae*	[78]

**Table 3:** Main advantages and disadvantages of dPCR technology.

Advantages
Absolute quantification, no need to rely on reference or standard for several applications
Sensitivity and accuracy, useful to detect rare and low copy number targets
Suitability for allelic variant detection
Applicable to complex mixtures and complex background
Resistance to PCR inhibitors
Linear response to number of copies
**Disadvantages**
More expensive compared to qPCR, although questionable
Limited dynamic range of detection
Problems with very large amplicons
More complex work-flow compared to qPCR
More expensive instrumentation compared to qPCR
